# Erythropoietin production by the kidney and the liver in response to severe hypoxia evaluated by Western blotting with deglycosylation

**DOI:** 10.14814/phy2.14485

**Published:** 2020-06-26

**Authors:** Yukiko Yasuoka, Takashi Fukuyama, Yuichiro Izumi, Yushi Nakayama, Hideki Inoue, Kengo Yanagita, Tomomi Oshima, Taiga Yamazaki, Takayuki Uematsu, Noritada Kobayashi, Yoshitaka Shimada, Yasushi Nagaba, Masashi Mukoyama, Tetsuro Yamashita, Yuichi Sato, Jeff M. Sands, Katsumasa Kawahara, Hiroshi Nonoguchi

**Affiliations:** ^1^ Department of Physiology Kitasato University School of Medicine Sagamihara Japan; ^2^ Division of Biomedical Research Kitasato University Medical Center Kitamoto Japan; ^3^ Department of Nephrology Kumamoto University Graduate School of Medicine Kumamoto Japan; ^4^ Department of Molecular Diagnostics Kitasato University School of Allied Health Sciences Sagamihara Japan; ^5^ Division of Internal Medicine Kitasato University Medical Center Kitamoto Japan; ^6^ Department of Biological Chemistry and Food Sciences Faculty of Agriculture Iwate University Morioka Japan; ^7^ Renal Division Department of Medicine Emory University School of Medicine Atlanta GA USA

**Keywords:** anemia, deglycosylation, erythropoiesis‐stimulating agents, erythropoietin, hypoxia

## Abstract

The detection of erythropoietin (Epo) protein by Western blotting has required pre‐purification of the sample. We developed a new Western blot method to detect plasma and urinary Epo using deglycosylation. Epo in urine and tissue, and erythropoiesis‐stimulating agents (ESAs) in urine were directly detected by our Western blotting. Plasma Epo and ESAs were not detected by direct application but were detected by our Western blotting after deglycosylation. The broad bands of Epo and ESAs were shifted to 22 kDa by deglycosylation except for PEG‐bound epoetin β pegol. The 22 kDa band from an anemic patient's urine was confirmed by Liquid Chromatography/Mass Spectrometry (LC/MS) to contain human Epo. Severe hypoxia (7% O_2,_ 4 hr) caused a 400‐fold increase in deglycosylated Epo expression in rat kidneys, which is consistent with the increases in both Epo gene expression and plasma Epo concentration. Immunohistochemistry showed Epo expression in nephrons but not in interstitial cells under control conditions, and hypoxia increased Epo expression in interstitial cells but not in tubules. These data show that intrinsic Epo and all ESAs can be detected by Western blot either directly in urine or after deglycosylation in blood, and that the kidney but not the liver is the main site of Epo production in control and severe hypoxia. Our method will make the tests for Epo doping and detection easy.

## Graphical abstract

The glycosylated Epo production by the kidney in response to severe hypoxia increased by 10–20 times. Deglycosylated Epo production by the kidney showed 400‐fold increase by severe hypoxia.

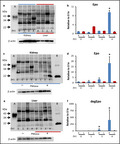

## 1 INTRODUCTION

Anemia is one of the most common diseases in humans (Lopez, Cacoub, MacDougall, & Peyrin‐Biroulet, 2016). Severe anemia and hypoxia stimulate erythropoietin (Epo) production by the kidney (Haase, 2013; Kobayashi et al., 2016; Koury & Haase, 2015; Koury, Bondurant, & Koury, 1988; Lacombe et al., 1988; Obara et al., 2008; Semenza, Koury, Nejfelt, Gearhart, & Antonarakis, 1991). The increase in Epo production is measured by the increases in serum and urine Epo concentrations and in Epo mRNA expression in the kidney (Chikuma, Masuda, Kobayashi, Nagao, & Sasaki, 2000; Eckardt, Dittmer, Neumann, Bauer, & Kurtz, 1990; Kobayashi et al., 2016; Koury et al., 1988; Lacombe et al., 1988; Obara et al., 2008; Pan et al., 2011; Semenza et al., 1991; Tan, Eckardt, Firth, & Ratcliffe, 1992). Serum or urine Epo concentrations have been measured by radio immunoassay (RIA) or enzyme‐linked immunosorbent assay (ELISA) using antibodies against Epo (Chikuma et al., 2000; Eckardt et al., 1990; Kobayashi et al., 2016; Lundby, Keiser, Siebenmann, Schaffer, & Lundby, 2014; Tan et al., 1992). However, Epo protein expression in the kidney or liver has not been measured accurately, since Western blotting of Epo has required pre‐purification of the sample. Higuchi, et al. reported glycosylated and deglycosylated human Epo (hEpo) by SDS‐PAGE and coomassie brilliant blue (CBB) staining (Higuchi, et al. 1992). Yamaguchi, et al, showed Western blotting of glycosylated and degycosylated Epos after the purification of samples by immunoaffinity chromatography (Yamaguchi et al., 1991). Kodama and colleagues reported chimeric chickens‐produced‐glycosylated and deglycosylated hEpos by Western blotting after partial purification of the samples by blue‐sepharose resin (Kodama et al., 2008). The World Anti‐Doping Agency (WADA) Technical Documents for Epo (TD2014EPO in TD2019INDEX) recommended the use of isoelectrical focusing (IEF) and/or SAR‐PAGE after enrichment for Epo through ultrafiltration, selective protein precipitation or immunopurification to detect Epo in the urine or serum/plasma (Heuberger et al., 2019; WADA EPO Working Group, 2019). Serum/urine Epo concentrations, and Epo mRNA and HIF1α/2α expressions in the kidney and liver have been used as a substitute for Epo protein expression in the kidney and liver (Chikuma et al., 2000; Eckardt et al., 1990; Kobayashi et al., 2016; Koury et al., 1988; Lacombe et al., 1988; Lundby et al., 2014; Obara et al., 2008; Pan et al., 2011; Semenza et al., 1991; Tan et al., 1992). However, the increase of kidney‐produced Epo has not been shown to increase to the same degree. This suggests the possibility that Epo production by the liver may have some role in the increase of Epo production in response to severe hypoxia (Haase, 2013; Heuberger et al., 2019; Koury & Haase, 2015).

The discovery of Epo led to the invention of erythropoiesis stimulating agents (ESAs) to treat anemic patients with chronic kidney disease (CKD) (Jacobs et al., 1985; Kalantar‐Zadeh, 2017; Miyake, Kung, & Goldwasser, 1977). ESAs have also been illegally used by athletes to improve physical activity, leading to tests for doping (Reichel, 2011). Although the details of tests for Epo doping are not known, WADA recommended the use of isoelectrical focusing (IEF) and/or SAR‐PAGE after enrichment for Epo (Heuberger et al., 2019; WADA EPO Working Group, 2019). ELISA or Liquid Chromatography/Mass Spectrometry (LC/MS) after the pre‐purification of urine is also useful. These recommendations clearly show that the detection of Epo by Western blotting required pre‐purification of the samples.

We have reported a new method of Western blot analysis that successfully detects kidney‐produced Epo (Yasuoka et al, 2018). We have reported that Epo is produced by the cortical nephrons in control conditions using in situ hybridization, immunohistochemistry and real‐time PCR with microdissected nephron segments. We also showed that Epo production by the intercalated cells of the collecting ducts is regulated by the renin‐angiotensin‐aldosterone system (Yasuoka et al., 2018). We modified our method to detect plasma and urinary Epo. In this study, we report a new Western blot method for the detection of Epo protein in the plasma or urine. Using our new method, we investigated the role of the kidney and liver for Epo production in response to severe hypoxia.

## 2 METHODS

### 2.1 Materials and animals

Male Sprague‐Dawley rats (Japan SLC, Hamamatsu, Japan) were used in our study. In the severe hypoxia experiments, rats were exposed to 7% O_2_ and 93% N_2_ for 1–4 hr, which is known to stimulate rapid Epo production and is closer to the conditions at the summit of Mount Everest (Chikuma et al., 2000; Eckardt et al., 1990; Grocott et al., 2009). For the detection of ESAs in plasma and urine, large doses of ESAs were administered to some rats through the vena cava, and plasma and urine were collected after 30 min from the aorta and bladder, respectively. Animal experiments were conducted in accordance with the Kitasato University Guide for the Care and Use of Laboratory Animals and were approved by the Institutional Animal Care and Use Committee (Approval No. 2018‐030, 25‐2). Blood and urine were collected from patients with CKD who received ESAs and from patients with severe anemia. Urine was concentrated using Vivaspin (GE Healthcare Bio‐Science AB, Sweden). Our protocols were checked and approved by the above committee and the Ethics Committee at Kitasato University Medical Center (25‐2, 2018032, 2019029). Informed consent was obtained from all patients.

### 2.2 Real‐time PCR in control and hypoxic rats

The renal cortex and liver were collected from control and hypoxic rats. RNA was extracted using the RNeasy Mini Kit (Qiagen, 74106) and Qiacube. cDNA was synthesized using a Takara PrimeScript™ II 1st strand cDNA Synthesis Kit (Takara, 6210). Real‐time PCR was performed using probes from Applied Biosystems and Premix Ex Taq (Takara, RR39LR). Probes were obtained from Applied Biosystems (Epo, Rn01481376_m1; HIF2α, Rn00576515_m1; HIF1α, Rn01472831_m1; PHD2, Rn00710295_m1, Thermo Fisher Scientific, USA). β‐actin (Rn00667869_m1) was used as an internal standard.

### 2.3 Western blot analysis

Western blot analysis was performed as described previously (Nonoguchi et al., 1995; Yasuoka et al., 2018). Protein was collected from the renal cortex and liver using CelLytic MT (Sigma‐Aldrich, C‐3228) plus protease inhibitor (Roche, 05892970001). Urine samples were obtained from rats injected with large doses of ESAs 30 min before the collection and from anemic patients. Plasma was obtained from rats injected with a large dose of ESAs and from patients with iron deficiency anemia or CKD. An anemic patient was treated by iron supplementation and blood was collected at severe and mild anemia and after complete recovery. Blood was also collected from CKD patients who were treated by the injection of epoetin β pegol and control subject. Informed consent was obtained from all patients. Urine samples were concentrated by Vivaspin (GE Healthcare Bio‐Science AB) and used for Western blot. Plasma samples were used directly or after deglycosylation as described below. Fifty to 80 µg of kidney or liver samples were used for SDS‐PAGE (10%–20% gradient gel, Cosmo Bio No. 414893, Tokyo, Japan). After SDS‐PAGE, proteins were transferred to a PVDF membrane (Immobilon‐P, Merck Millipore, IPVH00010) with 160 mA for 90 min. The membrane was blocked with 5% skim milk (Morinaga, Japan) for 60 min and incubated with the antibody against Epo (Santa Cruz, sc‐5290, 1:500–2,000) for 60 min at room temperature. After washing, the membrane was incubated with a secondary antibody (the goat anti‐mouse IgG (H + L) (Jackson ImmunoResearch Laboratories, 115‐035‐166, 1:5,000) for 60 min. Bands were visualized by the ECL Select Western Blotting Detection System (GE Healthcare Bio‐Science AB, RPN2235) and LAS 4000 (Fujifilm). The band intensity was normalized against that of β‐actin (MBL, M177‐3), which was measured after stripping and reprobing the membrane (stripping solution, Wako, RR39LR). In some experiments, another antibody against Epo (clone AE7A5, MAB2871, R & D Systems) was used to compare the specificity of the antibody. Clone AE7A5 and sc‐5290 were diluted with 5% milk (1:500).

### 2.4 Deglycosylation study

Since the Epo protein is a glycosylated protein, deglycosylation was performed. N‐glycosidase *F* (PNGase, Takara, 4450) was used as previously reported (Nonoguchi et al., 1995). In brief, a mixture of 7.5 μl of plasma, 2.5 μl of water, and 1 μl of 10% SDS was boiled for 3 min. Then, 11 μl of 2× stabilizing buffer was added, and 2 μl of PBS or PNGase was added. The samples were incubated in a water bath at 37°C for 15–20 hr. After incubation, the samples were spun down, and the supernatant was collected. For urine analysis, 7.5 μl–30 ml of urine was used either directly or after concentration by Vivaspin. To 10 μl of concentrated urine, 1 μl of 10% SDS was added and boiled for 3 min. The subsequent steps were the same as those performed for plasma. In the kidney and liver samples, 10 μl samples (50–80 µg protein) were treated in the same manner as urine. The 2× stabilizing buffer contained 62.5 mM Tris‐HCl (pH 8.6), 24 mM EDTA, 2% NP‐40 and 4% 2‐mercaptoethanol.

### 2.5 Plasma Epo concentration measurements

Plasma and urine were collected from control and hypoxic rats. Plasma, serum, and urine were also collected from patients with renal anemia treated with ESAs or from patients with iron‐deficient anemia. Plasma, serum, and urine Epo concentrations were measured by CLEIA (SRL, Tokyo, Japan, using Access Epo by Beckman Coulter, Brea, USA).

### 2.6 Immunohistochemistry of Epo production sites

Immunohistochemistry (IHC) of Epo expression was performed in control and severe hypoxic rats as previously reported (Nagai et al., 2014; Yasuoka et al., 2018; Yasuoka, Sato, Healy, Nonoguchi, & Kawahara, 2015). A polyclonal antibody against the same sequences as sc‐5290 was used, namely, sc‐7956. Images were obtained using an optical microscope (Axio Imager. M2, Carl Zeiss, Oberkochen, Germany) with a digital camera (AxioCam 506, Carl Zeiss). Captured images were analyzed using an image analysis system (ZEN 2, Carl Zeiss).

### 2.7 LC/MS analysis of band from Western blot

The 22 kDa band of the Western blot was excised and subjected to LC/MS as previously reported (Takahashi, Kawamura, Yamashita, & Uemura, 2012). Negative staining was used to detect deglycosylated recombinant Epo. The negatively stained protein bands were excised from the SDS‐PAGE gel, and in‐gel tryptic digestion was carried out using ProteaseMAX reagent (Promega, WI, USA) according to the manufacturer's protocol. The peptides were separated by L‐column 2 ODS (3 μm, 0.1 × 150 mm, CERI, Tokyo, Japan) at a flow rate of 500 nl/min using a linear gradient of acetonitrile (5% to 45%). Nano‐LC–MS/MS analyses were performed with an LTQ‐Orbitrap XL mass spectrometer (Thermo Fisher Scientific, MA, USA) as previously described (Takahashi et al., 2012).

### 2.8 Statistical analyses

Statistical analyses were performed using Excel Statics (BellCurve, Tokyo, Japan). Statistical significance was analyzed using non‐parametric analysis by the Kruskal–Wallis test and multiple comparisons by the Shirley–Williams test. *p* < .05 was considered statistically significant. Results were expressed as mean ± standard error of mean (*SEM*).

## 3 RESULTS

### 3.1 Detection of Epo protein

**FIGURE 1:**
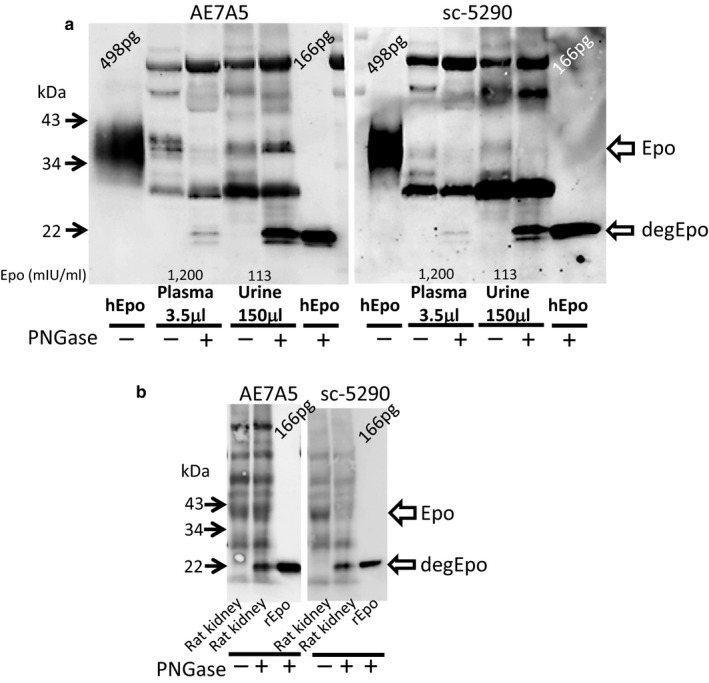
Comparison of AE7A5 and sc‐5290. (a) Plasma and concentrated urine from anemic patients were used for Western blotting with or without deglycosylation. Although both clone AE7A5 (1:500) and sc‐5290 (1:500) recognize Epo and deglycosylated Epo (degEpo) at 36–40 kDa and 22 kDa, respectively, the specificity of sc‐5290 was better than that of clone AE7A5, especially after deglycosylation. (b) The kidney cortex from hypoxic rats was used for Western blotting. Although a 36–40 kDa band of Epo by sc‐5290 became pale after deglycosylation, the same band by clone AE7A5 remains strong after deglycosylation. hEpo; recombinant human Epo, rEpo; recombinant rat Epo

**FIGURE 2:**
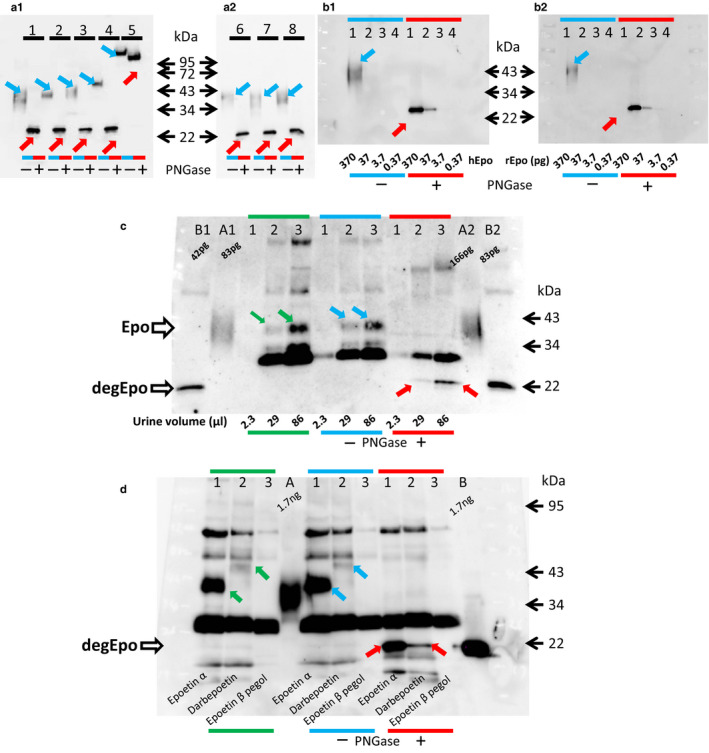
Detection of Epo and ESAs in urine by Western blotting. a1. Expression of recombinant human Epo and ESAs detected by Western blotting. Recombinant hEpo shows a broad band at 34–43 kDa. Epoetin α and β, darbepoetin and epoetin β pegol gradually increased in size. Deglycosylation shifted all human Epo and ESAs to 22 kDa except PEG‐bound epoetin β pegol. Lane 1: recombinant hEpo; Lane 2, epoetin α; lane 3, epoetin β; lane 4, darbepoetin; and lane 5, epoetin β pegol. The left and right lanes of each peptide are without (blue arrow in blue line) and with (red arrow in red line) deglycosylation, respectively. a2. Expression of rat (lane 6), mouse (lane 7) and human Epo (lane 8). Rat, mouse and human Epo showed the same expression at 34–43 kDa (blue arrow in blue line), and deglycosylation shifted all bands to 22 kDa (red arrow in red line). (b1–2) Expression of recombinant human (b1) and rat (b2) Epo in glycosylated (blue arrow in blue line) and deglycosylated forms (red arrow in red line). The detection limits of glycosylated and deglycosylated human Epo were 370 and 37 pg, respectively. (c) Detection of intrinsic Epo in human urine. Urine from an anemic patient was applied to the Western blot: 2.3, 29 and 86 μl samples of urine (Epo concentration 152 mIU/ml) were concentrated by Vivaspin and used in lanes 1, 2, and 3, respectively. Epo protein bands were observed dose‐dependently at 36–40 kDa (green and blue line in Figure 2c). Deglycosylated Epo was observed in more than 29 μl of urine (red line in Figure 2c). A, B: glycosylated and deglycosylated recombinant hEpo, respectively. Green line, direct application; blue line, incubation with deglycosylation buffer; and red line, after deglycosylation. (d) Detection of ESAs in rat urine. Male *SD* rats (200 g) were injected with epoetin α (600 μg), darbepoetin (4.5 μg), or epoetin β pegol (3.8 μg), and urine was obtained after 30 min. The plasma Epo concentrations of each rat were 37,800, 29,400 and 527 mIU/ml for epoetin α, darbepoetin and epoetin β pegol, respectively. The direct analysis of urine (5 μl) showed a clear and broad band of epoetin α at 36–40 kDa (lane 1 and green arrow in green line). The band of darbepoetin was pale (lane 2 and blue arrow in blue line) and that of epoetin β pegol was not observed (lane 3 in green line). The band of darbepoetin became slightly clearer after incubation of urine with deglycosylation buffer (lane 2 and blue arrow in blue line). The bands of epoetin α and darbepoetin were shifted to 22 kDa (lanes 1 and 2 and red arrows in red line). The deglycosylated band of darbepoetin (lane 2 and an arrow in red line) was clearer than the glycosylated band. Since the rat urine samples were very small, the urine Epo concentration was not measured. (a and b) glycosylated and deglycosylated rat Epo, respectively

We reported that our Western blot recognized hypoxic rat kidney Epo protein and the deglycosylated protein at 34–43 and 22 kDa, respectively (Yasuoka et al., 2018). AE7A5 showed higher sensitivity than sc‐5290 for the detection of glycosylated Epo. However, the specificity of sc‐5290 was better than that of AE7A5, especially after the deglycosylation of urine and kidney (Figure 1a and b). The band at 36–40 kDa by sc‐5290 almost disappeared, whereas the same band by clone AE7A5 became much stronger after deglycosylation, suggesting that clone AE7A5 recognizes Epo and non‐Epo bands at 36–40 kDa. ESAs were also detected by Western blot, and deglycosylation caused a shift of the bands to 22 kDa, except for that of epoetin β pegol (Figure 2a1,2a 2). The deglycosylated band at 22 kDa showed a 10 times lower limit of detection than the non‐deglycosylated band at 34–43 kDa (Figure 2b1, 2b2). The size of Epo is largely dependent on the amount of recombinant human Epo (Figure S1).

### 3.2 Detection of Epo protein and ESAs in urine

The direct analysis (green line) and incubation with deglycosylation buffer (blue line) of anemic patient's urine both volume‐dependently showed an Epo protein band at 36–40 kDa (Epo in lanes 2 and 3 in Figure 2c). Deglycosylation (red line) shifted the bands to 22 kDa (degEpo in red line of Figure 2c). Epoetin α (lane 1) and darbepoetin (lane 2) were detected by the direct application of rat urine after bolus injection (lanes 1 and 2 and arrows in green and blue line in Figure 2d). Epoetin β pegol (lane 3) was not detected, probably due to its limited excretion into the urine (Figure 2d). The bands of epoetin α and darbepoetin were shifted to 22 kDa by degycosylation (degEpo, red arrows in lanes 1 and 2 in red line of Figure 2d).

### 3.3 Detection of Epo protein and ESAs in plasma

**FIGURE 3:**
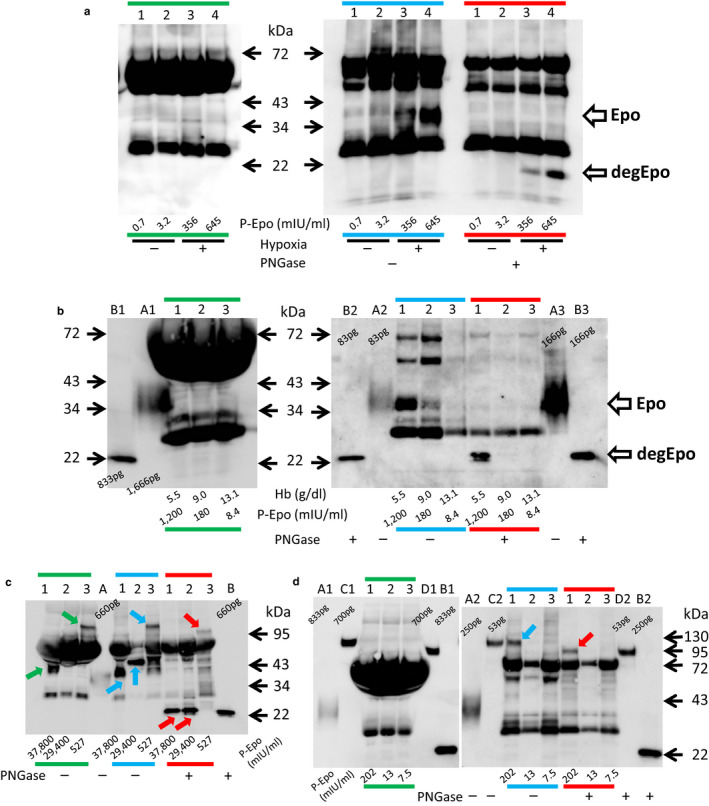
Detection of Epo and ESAs in plasma. (a) Detection of intrinsic rat Epo in control and hypoxic rats. Although no bands were observed by in the direct analysis of plasma (2 μl) (green line), the incubation of plasma from hypoxic rats (7 μl) with deglycosylation buffer (Epo in blue line) resulted in the appearance of Epo bands at 34–43 kDa, which were shifted to 22 kDa by deglycosylation (degEpo in red line). Lanes 1–2, control rats. Lanes 3–4, hypoxic rats. The plasma Epo concentrations in each rat were 0.7, 3.2, 356, and 645 mIU/ml, respectively. The green, blue, and red lines show direct application and incubation with deglycosylation buffer without and with PNGase, respectively. (b) Detection of intrinsic hEpo in the plasma of a patient with severe anemia. Plasma was obtained under severe and mild anemia and after recovery (plasma hemoglobin levels were 5.5, 9.0, and 13.1 g/dl, respectively). No bands were observed with the direct analysis of plasma (2 μl) (green line in Figure 3b). Incubation of plasma (5 μl) with deglycosylation buffer revealed the band at 36–40 kDa only under anemic conditions, and the bands were shifted to 22 kDa (Epo in lane 1 in blue line and degEpo in lane 1 in red line of Figure 3b). Plasma Epo concentrations were 1,200, 180, and 8.4 mIU/ml, respectively. (a, b); glycosylated and deglycosylated recombinant human Epo, respectively. (c) Detection of ESAs in rats injected with large doses of ESAs. Male *SD* rats were injected with epoetin α, darbepoetin and epoetin β pegol as described in Figure 2d, and blood was obtained after 30 min. The bands of epoetin α and epoetin β pegol were observed by the direct analysis of plasma (2 μl) (green arrows in lanes 1 and 3 in green line of Figure 3c), while the band of darbepoetin was obscured by a non‐specific band (lane 2 in green line of Figure 3c). Incubation of plasma (5 μl) with deglycosylation buffer reduced the non‐specific band, and the band of darbepoetin became clear (blue arrow in lane 2 in blue line of Figure 3c). The bands of epoetin α and darbepoetin shifted to 22 kDa, while the band of epoetin β pegol was slightly reduced in size (red arrows in lanes 1–3 in red line of Figure 3c). A, B; glycosylated and deglycosylated recombinant rat Epo, respectively. (d) Detection of plasma ESA in patients. Plasma samples from patients treated with epoetin β pegol and control subject was subjected to Western blotting. No bands were observed by the direct analysis of plasma (2 μl) (green line in Figure 3d). The incubation of plasma (3.5 μl) with deglycosylated buffer revealed the band corresponding to epoetin β pegol in patient 1 at 95–130 kDa (blue arrow in lane 1 in blue line of Figure 3d). The band was shifted to 80–95 kDa by deglycosylation (red arrow in lane 1 in red line of Figure 3d). The plasma Epo concentrations of each subject were 202, 13, and 7.5 mIU/ml, respectively. Patient 1:76 y.o., male, 47.1 kg, serum creatinine 11.93 mg/dl, Hb 8.2, epoetin β pegol injection 3 days before. Patient 2:74 y.o., female, 53.5 kg, serum creatinine 3.15 mg/dl, Hb 10.8 g/dl, epoetin β pegol injection 28 days before. Patient 3:65 y.o., male, 62 kg, serum creatinine 0.73 mg/dl, Hb 15.1 g/dl, no injection. A, B: glycosylated and deglycosylated recombinant human Epo, respectively. C, D: glycosylated and deglycosylated epoetin β pegol, respectively

The direct analysis of plasma from control and hypoxic rats by Western blotting showed no band (green line in Figure 3a). Incubation of the plasma with deglycosylation buffer showed broad bands at 34–43 kDa in 4 hr hypoxic rats but not in control rats (Epo, lanes 3 and 4 in blue line of Figure 3a, respectively). Deglycosylation shifted the broad band at 34–43 kDa to 22 kDa (degEpo, lanes 3 and 4 in red line of Figure 3a, respectively). Next, direct analysis of plasma from an anemic patient also showed no band (green line in Figure 3b). Incubation of the plasma with deglycosylation buffer showed a broad band at 36–40 kDa only in the case of severe anemia (Epo, lane 1 in blue line of Figure 3b). The partial recovery of anemia caused a faint band, and complete recovery revealed no broad band at 36–40 kDa (lanes 2 and 3 in blue line of Figure 3b, respectively). Deglycosylation caused an intense band in anemia, and partial recovery of anemia caused a very faint band at 22 kDa (degEpo, lanes 1 and 2 in red line of Figure 3b, respectively). No band was observed at 22 kDa after complete recovery.

The detection of ESAs in plasma was tested in rats after the intravenous injection of large doses of ESAs. The plasma Epo concentration was more than 100 times higher than under severe hypoxia. In this condition, epoetin α and epoetin β pegol were detected by the direct analysis of plasma (green arrows and line in Figure 3c). The band of darbepoetin overlapped with the non‐specific band, which was removed by the incubation of plasma with deglycosylation buffer (lane 2, blue arrows and line in Figure 3c). The bands of epoetin α and darbepoetin were shifted to 22 kDa by deglycosylation (lanes 1 and 2, red arrows and line in Figure 3c). The band of epoetin β pegol shifted from 95–120 to 80–95 kDa (lane 3, red arrow in Figure 3c). In contrast, no band representing epoetin β pegol was detected by the direct analysis of plasma from anemic CKD patients (green line in Figure 3d). The incubation of plasma with deglycosylation buffer induced the appearance of a band at 95–120 kDa (blue arrow and line in Figure 3d), which was shifted to 80–95 kDa by deglycosylation (red arrow and line in Figure 3d).

### 3.4 Detection of Epo protein by LC/MS

**FIGURE 4:**
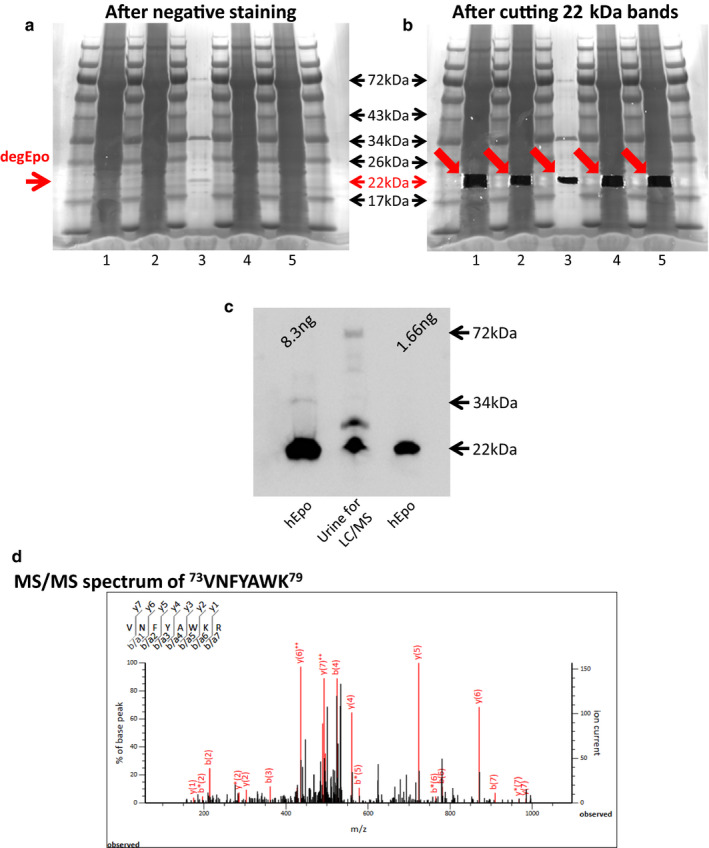
LC/MS detection of recombinant and intrinsic Epo. (a and b) Deglycosylated recombinant hEpo and urine samples of anemic patients were subjected to SDS‐PAGE and negative staining. The 22 kDa bands were excised and subjected to LC/MS. Although the recombinant hEpo (8.3 ng) was seen by negative staining (lane 3), no 22 kDa band was observed in the human urine samples (concentrated from 3.1 ml of urine, lanes 1, 2, 4, and 5). (c) Western blotting of urine sample used for LC/MS analysis. 12.5 µl of concentrated urine was used in Figure c and 15 µl of concentrated urine was used for Figure a. (d) MS/MS spectra of recombinant human Epo peptides: ^73^VNFYAWK^79^. The red line shows the expected peptides, and the black line shows the observed peptides

To confirm that the band at 22 kDa is Epo protein, the 22 kDa band of recombinant human Epo, and anemic patient's urine were excised and analyzed by LC/MS (Figure 4a and b). Seven and three peptide sequences of human Epo protein (sequence coverage 20% and 12%) were identified in the sample of recombinant human Epo and anemic patients, respectively (Table 1). Recombinant rat Epo was also identified by LC/MS (Table 1). The size of human and rat Epo by LC/MS was 21.6 Da, which is compatible with the size by Western blotting.

**TABLE 1:** (A–C) LC/MS analysis of the 22 kDa band of recombinant human Epo (8.3 ng), concentrated human urine from anemic patients and recombinant rat Epo (4.4 pg), respectively. Matched peptides are shown in underline. (D–F) Detailed LC/MS data on matched peptides of recombinant human Epo (D), human urine sample (E), and recombinant rat Epo (F)

A Human (Recombinant): Coverage 20%					
1	MGVHECPAWL	WLLLSLLSLP	LGLPVLGAPP	RLICDSRVLE	RYLLEAKEAE
51	NITTGCAEHC	SLNENITVPD	TKVNFYAWKR	MEVGQQAVEV	WQGLALLSEA
101	VLRGQALLVN	SSQPWEPLQL	HVDKAVSGLR	SLTTLLRALG	AQKEAISPPD
151	AASAAPLRTI	TADTFRKLFR	VYSNFLRGKL	KLYTGEACRT	GDR
B Human urine: Coverage 12%					
1	MGVHECPAWL	WLLLSLLSLP	LGLPVLGAPP	RLICDSRVLE	RYLLEAKEAE
51	NITTGCAEHC	SLNENITVPD	TKVNFYAWKR	MEVGQQAVEV	WQGLALLSEA
101	VLRGQALLVN	SSQPWEPLQL	HVDKAVSGLR	SLTTLLRALG	AQKEAISPPD
151	AASAAPLRTI	TADTFRKLFR	VYSNFLRGKL	KLYTGEACRT	GDR
C Rat (recombinant): Coverage 16%					
1	MGVPERPTLL	LLLSLLLIPL	GLPVLCAPPR	LICDSRVLER	YILEAKEAEN
51	VTMGCAEGPR	LSENITVPDT	KVNFYAWKRM	KVEEQAVEVW	QGLSLLSEAI
101	LQAQALQANS	SQPPESLQLH	IDKAISGLRS	LTSLLRVLGA	QKELMSPPDA
151	TQAAPLRTLT	ADTFCKLFRV	YSNFLRGKLK	LYTGEACRRG	DR

D				
Peptide	Observed	Mr(expt)	Mr(calc)	ppm
^73^VNFYAWK^79^	464.2393	926.4640	926.4650	‐1.14
^73^VNFYAWKR^80^	542.2902	1082.5659	1082.5661	‐0.23
^131^SLTTLLR^137^	402.2527	802.4909	802.4912	‐0.37
^159^TITADTFR^166^	462.7429	923.4712	923.4713	‐0.09
^159^TITADTFRK^167^	526.7903	1051.5660	1051.5662	‐0.19
^171^VYSNFLR^177^	449.7426	897.4706	897.4708	‐0.26
^182^LYTGEACR^189^	485.2262	968.4378	968.4386	‐0.73
E				
Peptide	Observed	Mr(expt)	Mr(calc)	ppm
^159^TITADTFRK^167^	526.7901	1051.5656	1051.5662	‐0.53
^171^VYSNFLR^177^	449.7425	897.4705	897.4708	‐0.39
^182^LYTGEACR^189^	485.2267	968.4387	968.4386	0.20
F				
Peptide	Observed	Mr(expt)	Mr(calc)	ppm
^72^VNFYAWKR^79^	542.2902	1082.5658	1082.5661	‐0.34
^158^TLTADTFCK^166^	528.7545	1055.4945	1055.4958	‐1.20
^170^VYSNFLR^176^	449.7427	897.4709	897.4708	0.031
^181^LYTGEACR^188^	485.2262	968.4378	968.4386	‐0.73

### 3.5 Epo mRNA and protein expression in hypoxia

**FIGURE 5:**
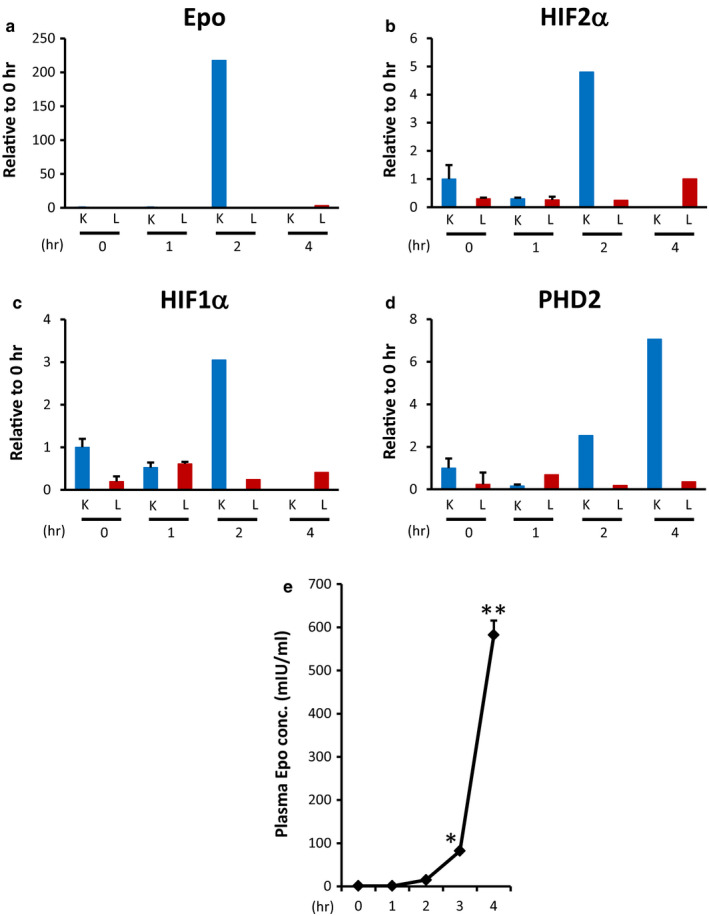
Effects of hypoxia on Epo mRNA expression in the kidney and liver. (a–d) Effects of severe hypoxia on the mRNA expression of Epo (a), HIF1α (b), HIF2α (c), and PHD2 (d) in the kidney and liver. Severe hypoxia increased HIF1α, HIF2α, and Epo mRNA expression after 2 hr in the kidney, and PHD2 mRNA expression in the kidney reached its maximum at 4 hr (Figure 5a–d). In contrast, Epo, HIF1α, HIF2α, and PHD2 mRNA expression in the liver did not change by hypoxia. *n* = 3–4 (Epo), *n* = 3 (HIF2α, HIF1α), *n* = 3–4 (PHD2), K0, K1, K2, and K4; zero time, 1, 2, and 4 hr after the induction of hypoxia in the kidney. L0, L1, L2, and L4: same time course in the liver. (e) Changes in plasma Epo concentration during severe hypoxia. The plasma Epo concentration significantly increased after 3 hr. 0 hr 1.1 ± 0.5, 1 hr: 1.1 ± 0.1, 2 hr: 14.9 ± 0.8, 3 hr: 82.0 ± 5.6, 4 hr: 582 ± 33.5 mIU/ml) *n* = 3–7. * *p* < .05, **p < .001 using the Kruskal–Wallis test and multiple comparisons by the Shirley–Williams test

**FIGURE 6:**
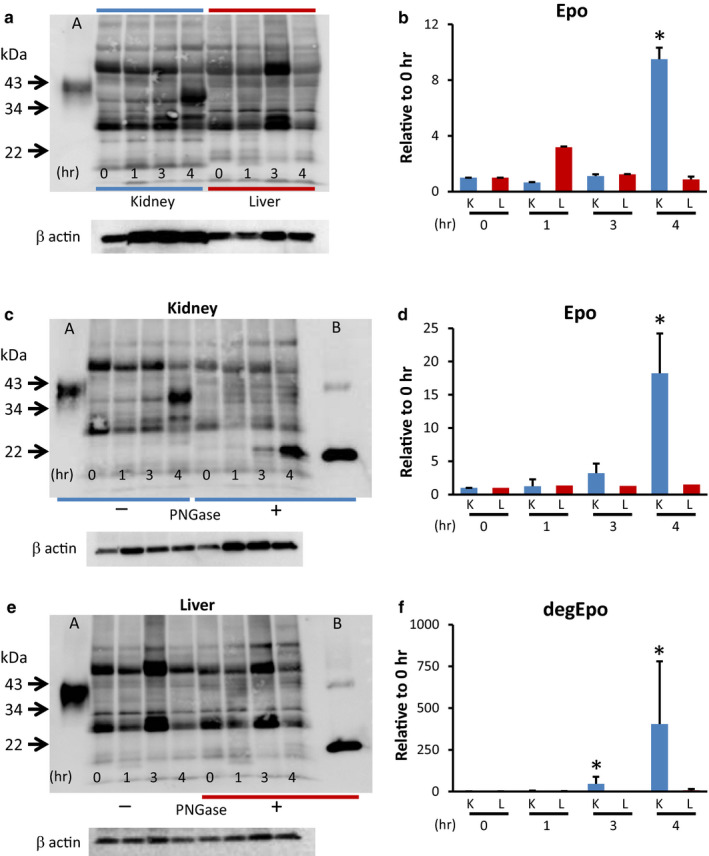
Effects of hypoxia on Epo protein expression in the kidney and liver. (a, c, e) Western blot analysis of Epo expression in the kidney and liver. A typical gel is shown in Figure a, c, and e, and the analyzed data are shown in Figure b, d, and f. Severe hypoxia increased Epo protein expression in the kidney at 4 hr by 10‐fold but did not increase in the liver (a, b). *n* = 4, * *p* < .05. Western blot analysis of Epo protein expression after deglycosylation in the kidney (c) and the liver (e). Glycosylated Epo protein expression increased by 20‐fold after 4 hr (d). *n* = 6, * *p* < .05. Deglycosylated Epo expression was observed from zero time to 4 hr. The expression increased by 400‐fold after 4 hr (f), which was close to the changes in the plasma Epo concentration (Figure 5e). In contrast, Epo protein expression in liver did not increase under hypoxia (e and f). *n* = 6, * *p* < .05 using the Kruskal–Wallis test and multiple comparisons by the Shirley–Williams test

Epo mRNA and protein expression in the kidney and liver in hypoxia were examined in rats. HIF1α, HIF2α, and Epo mRNA expression in the kidney reached a maximum at 2 hr after hypoxia, and PHD2 mRNA expression in the kidney reached its maximum at 4 hr (Figure 5a–d). Epo mRNA showed a 200‐fold increase in the kidney with no changes in the liver (Figure 5a). The plasma Epo concentration showed a 529‐fold increase at 4 hr compared with zero time (Figure 5e). Epo protein expression in the kidney reached its maximum at 4 hr, while the changes in Epo protein expression in the liver were small (Figure 6a and b). Usual Western blot showed an approximately 10‐fold increase in Epo protein expression in the kidney (Figure 6a and b). Incubation of the kidney samples with deglycosylation buffer without PNGase made the bands clear and the increase of Epo protein expression reached a 20‐fold increase (Figure 6c and d). In contrast, deglycosylated Epo protein expression showed an approximately 400‐fold increase (Figure 6c and f), which is very close to the changes in plasma Epo concentration. A very faint band of deglycosylated Epo was observed in the hypoxic liver (Figure 6e and f).

### 3.6 Immunohistochemical Epo protein expression

**FIGURE 7:**
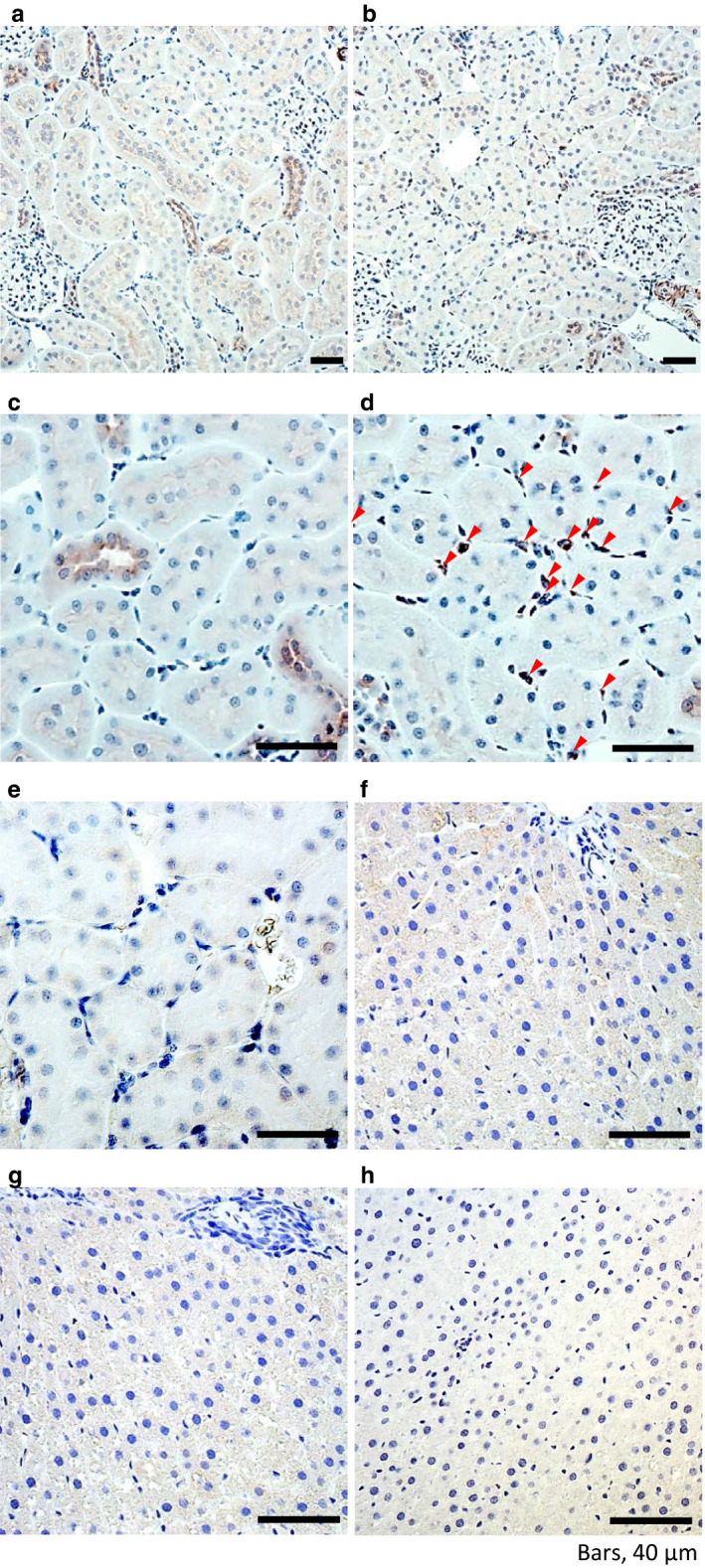
Immunohistochemical analysis of Epo protein expression in the kidney. Epo protein was observed in proximal and distal tubules at 21% O_2_ (a and c). Severe hypoxia (7% O_2_, 4 hr) increased Epo protein expression in the interstitial cells (arrowhead) while slightly decreasing the expression in the tubules (b and d). Negative control showed no staining (e). In contrast, no staining was seen in control, hypoxic, and negative control liver (f, g, and h, respectively)

Immunohistochemistry showed that renal proximal and distal tubules in the cortex were weakly stained under basal conditions (proximal tubules < thick ascending limbs, distal convoluted tubules) (Figure 7a and c). Severe hypoxia caused increased Epo staining of the interstitial cells around proximal tubules in the deep cortical area but decreased staining in tubular cells, as in our previous report (Yasuoka et al., 2018) using in situ hybridization (Figure 7b and d). The negative control showed no staining (Figure 7e). Immunohistochemistry of control and hypoxic liver showed no staining, as well as the negative control (Figure 7f–h, respectively).

## 4 DISCUSSION

We detected Epo protein and ESAs by the combination of usual Western blotting and LS/MS without pre‐purification of the samples for the first time. Urinary Epo and ESAs were detected by direct application of urine to SDS‐PAGE. However, intrinsic Epo and ESAs in plasma could not be detected even by our Western blot. The incubation of plasma in deglycosylation buffer resulted in the appearance of bands at 34–43 kDa, and deglycosylation caused a shift of those bands to 22 kDa, except for that of epoetin β pegol (CERA). LC/MS analysis of the 22 kDa band from an anemic patient's urine confirmed the presence of human Epo.

Since WADA recommended use of clone AE7A5 for Western blot analysis, we compared the sensitivity and specificity of sc‐5290 with that of clone AE7A5. Clone AE7A5 showed higher sensitivity than sc‐5290 for the detection of glycosylated Epo. However, sc‐5290 showed higher specificity for the detection of deglycosylated Epo. We think that sc‐5290 has higher specificity than clone AE7A5 for the detection of Epo and deglycosylated Epo.

One of the findings of our new method is that the detection limit of Epo protein is increased by deglycosylation. The detection limit of glycosylated and deglycosylated recombinant human and rat Epo was 370 and 37 pg, respectively (Figures 2b1 and 2b2). Therefore, deglycosylation increased the detection limit of Epo by 10 times. Accurate quantitative estimates of Epo can be obtained by measuring deglycosylated Epo. Although Epo is detected directly in the urine, the estimation of deglycosylated Epo in the urine would be more accurate.

Our method will make the tests for Epo doping easy. Currently, Epo doping is detected by IEF and/or SAR‐PAGE or LC/MS after pre et al., 2019; Reichel, 2011; WADA EPO Working Group, 2019). The report by Heuberger et al. examined the sensitivity and specificity of the detection of Epo using SAR‐PAGE and isoelectric focusing methods. The difference between the positive sample and the negative control is very small. Therefore, the conclusion regarding negative or positive is largely dependent on the evaluator's judgment. In contrast, our method is simple and the specificity is very high. Our method does not require any pre‐purification of the samples. Concentrated urine can be used directly for Western blotting. Blood samples should be deglycosylated to reduce non‐specific bands. Intrinsic Epo and ESAs are distinguished simply by band size. To completely confirm the presence of ESAs, cut gels should be checked by LC/MS. More than 1–2 ng of Epo was required to detect Epo by LC/MS, while the detection limit of Epo by our Western blotting is 3.7–37 pg. Since plasma or serum contains a lot of proteins, concentrated plasma results in a very high osmolality and is difficult to use for Western blotting. In contrast, urine usually has no protein except in patients with proteinuria, so concentrated urine can be used for Western blotting.

The detection of deglycosylated Epo expression by the kidney gave the answer to the sites of Epo production in response to severe hypoxia/anemia. Since the increase in Epo production in the kidney was not high enough compared to the changes in plasma Epo concentration and gene expression in the kidney, liver participation has been suggested (Fried, 1972; Haase, 2013; Kobayashi et al., 2016; Koury & Haase, 2015; Koury et al., 1988). The difficulty of Epo protein detection by Western blot was the main reason. We showed that deglycosylation increased the sensitivity of Epo detection by 10 times. Deglycosylated Epo expression showed a 400‐fold increase, which is very close to the change of Epo concentration in plasma. Deglycosylated Epo expression in the hypoxic liver was very low. The increases of HIF1α and HIF2α mRNA expression as well as Epo mRNA were observed in the hypoxic kidney but not in the hypoxic liver. The increase of PHD2 mRNA expression and a large decrease of Epo mRNA expression were observed in the kidney 4 hr after hypoxia. HIF2α has a key role for Epo production and PHD2 has a key role for the degradation of Epo (Lee & Percy, 2011; Paliege et al., 2010; Rosenberger, 2002). These data clearly show that the kidney, but not the liver, is the main site of Epo production in response to severe hypoxia. Although plasma Epo is very low in normal rats and humans, control rat kidneys showed deglycosylated Epo production, and immunohistochemistry showed Epo production in the cortical nephrons. Mujais and colleagues reported Epo mRNA expression in renal tubules using microdissected nephron segments in cobalt chloride‐injected rats (Mujais, Beru, Pullman, & Goldwasser, 1999). We have previously shown that fludrocortisone stimulated Epo production by the intercalated cells of the collecting ducts (Yasuoka et al., 2018). Our immunohistochemistry also showed that kidney interstitial cells respond to severe hypoxia by producing Epo. Yamamoto and colleagues showed that the site of Epo production by severe anemia is the interstitial cells using EPO promoter‐driven GFP expression (Pan et al., 2011; Obara et al., 2008). Since 27 kDa GFP goes into the nucleus, they may have overestimated the role of Epo production by interstitial cells in severe anemia. The cytoplasm of interstitial cells is very pale, so Epo production by interstitial cells under hypoxic conditions may not be as strong as expected. These data show that kidney nephrons produce Epo under control conditions and that kidney interstitial cells produce Epo in response to severe hypoxia or anemia. We examined the kidneys and liver of male rats. Since the sex difference in response to hypoxia is known, the effect of hypoxia will need to be examined in female rats in future studies (Suresh, Rajvanshi, & Noguchi, 2020).

In conclusion, our data showed that Epo protein can be detected in urine and tissue samples by direct Western blot analysis and in blood after deglycosylation. Our data also showed that the kidneys have dual Epo production systems, low production by the nephron under normal conditions, and hypoxia or anemia‐induced high production by the interstitial fibroblast‐like cells, and that the kidney but not the liver is the main site of Epo production in response to hypoxia or anemia. Our method will make testing for Epo doping and detection easy.

## CONFLICT OF INTEREST

The authors have no financial conflict of interest to declare.

## AUTHOR CONTRIBUTIONS

YY, YI, KK, and HN designed the research; YY, YN, HI, YoS, YN, and HN performed the animal research; YI, TF, KY, TU, and HN performed Western blot analysis; YY, TO, YuS, and KK performed IHC, TF, TaY, NK, and HN performed RNA extraction and PCR; YI and HN performed the statistical analyses; and TeY performed LC/MS. MM, YuS, and JMS advised on the experimental design and data interpretation.

## Supporting information



Figure S1
