# Supplementary figures and images for: Erythropoietin production by the kidney and the liver in response to severe hypoxia evaluated by Western blotting with deglycosylation
Source: Physiol Rep. 2020 Jun 26;8(12):e14485. doi: 10.14814/phy2.14485 (PMC7319944; doi:10.14814/phy2.14485)

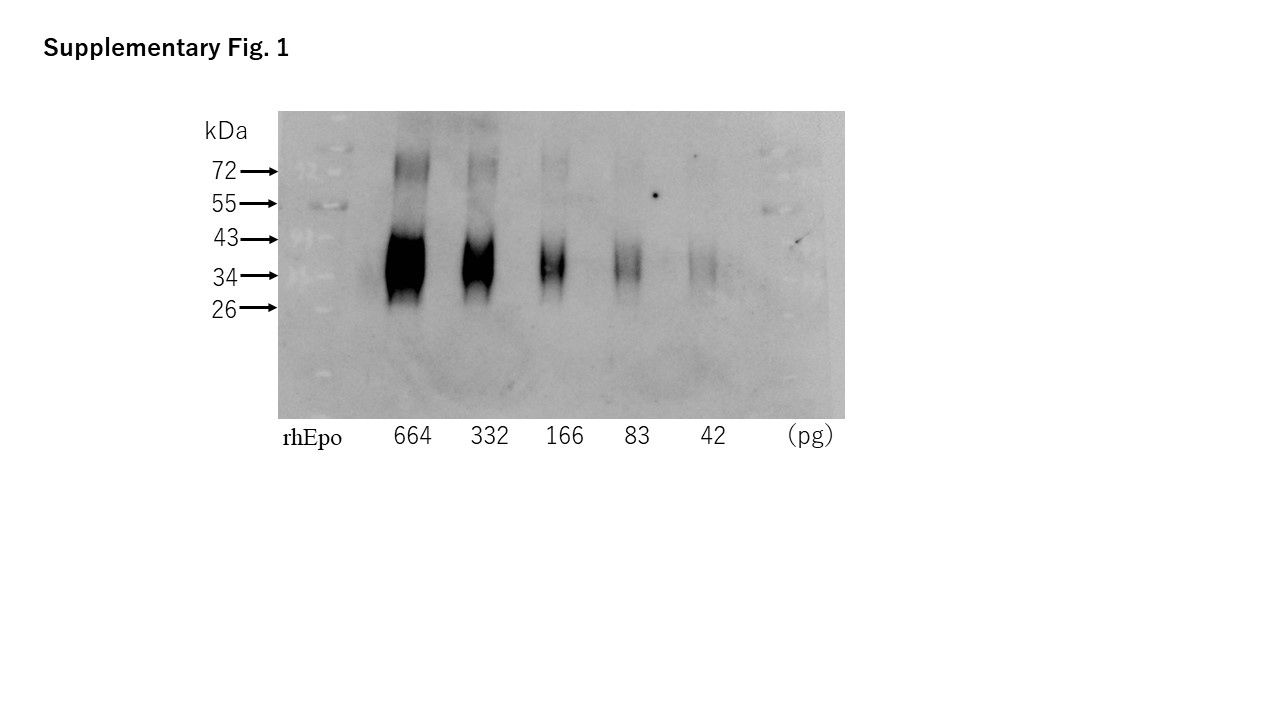

Supplement: Supplementary file 1 — Figure S1 [file PHY2-8-e14485-s001.jpg]
